# Supplementary material for: Structural basis for assembly and function of a diatom photosystem I-light-harvesting supercomplex
Source: Nat Commun. 2020 May 18;11:2481. doi: 10.1038/s41467-020-16324-3 (PMC7235021; doi:10.1038/s41467-020-16324-3)
Supplement: Supplementary file 1 — Supplementary Information [file 41467_2020_16324_MOESM1_ESM.pdf]

Supplementary Information

Structural basis for assembly and function of a diatom photosystem I-light harvesting supercomplex

Ryo Nagao, Koji Kato et al.

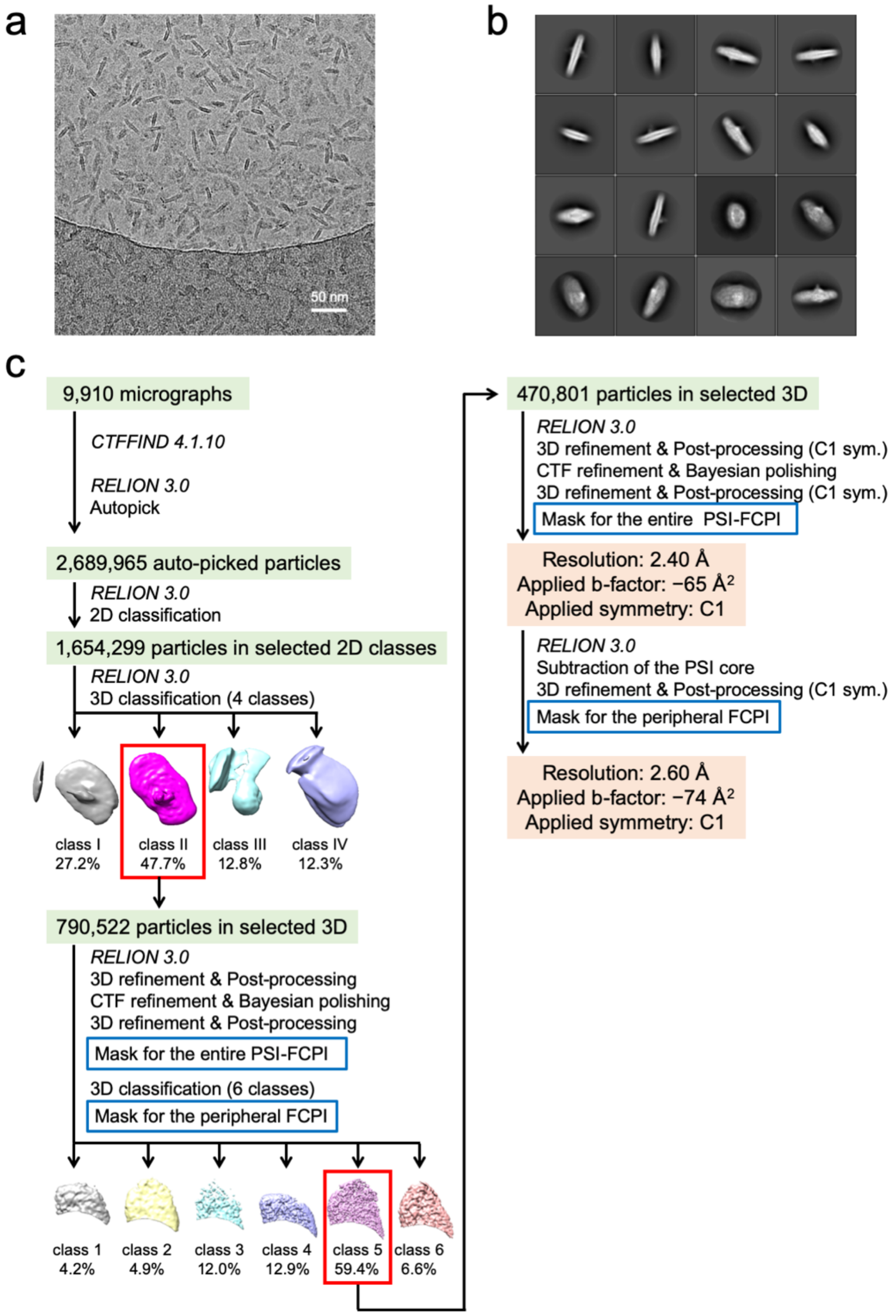

Supplementary Fig. 1. Cryo-EM data collection and processing for the PSI-FCPI supercomplex. a, A representative cryo-EM micrograph of the PSI-FCPI supercomplex. **b**, Representative 2D classes of the PSI-FCPI. The box size is 560 Å. **c**, A schematic flowchart showing the classification scheme for the PSI-FCPI. The overall PSI-FCPI structure was reconstructed at 2.40 Å resolution from 470,801 particles, whereas the peripheral FCPI structure from the subtracted particles was reconstructed at 2.60 Å resolution. See Methods section for more details.

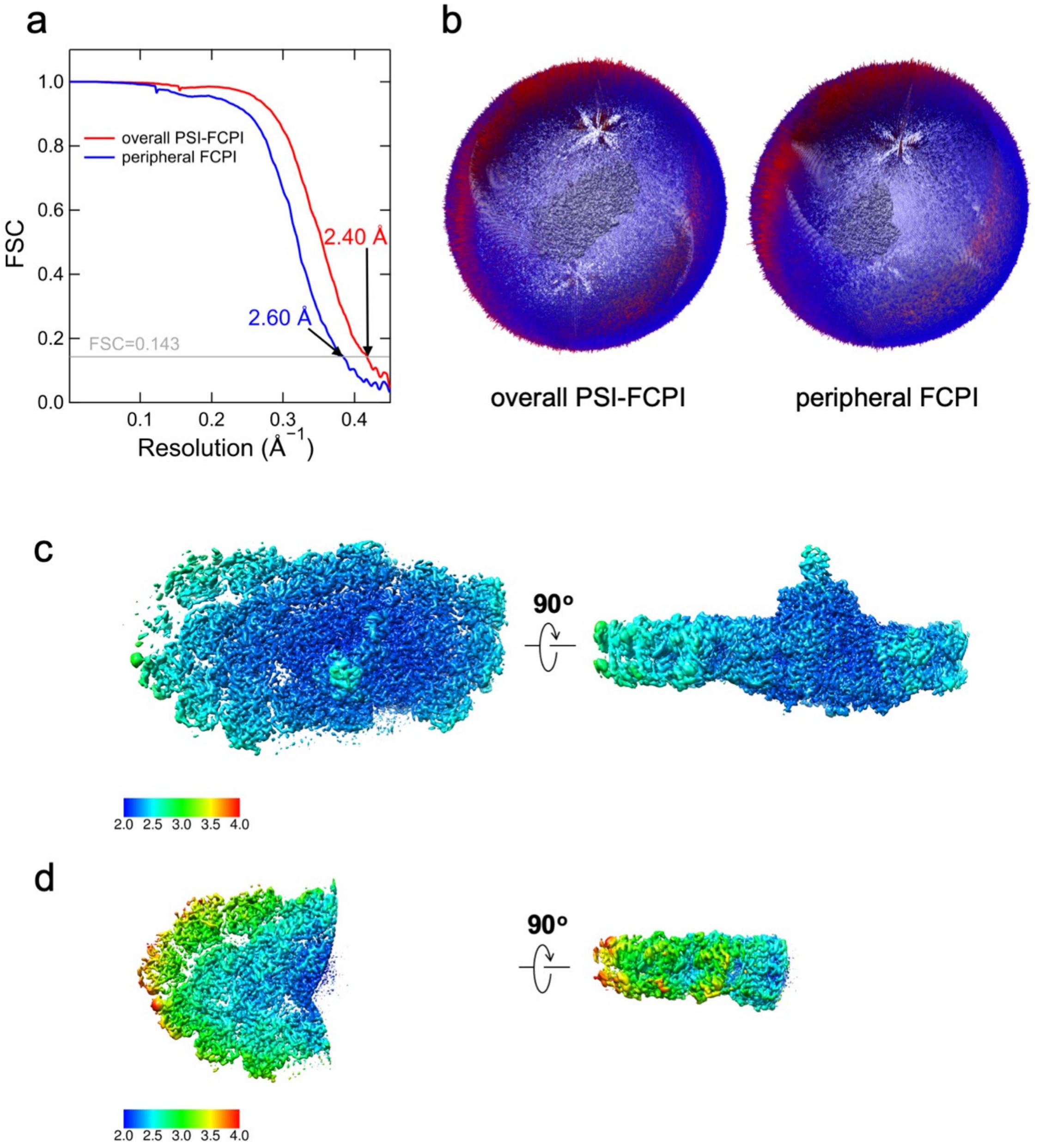

Supplementary Fig. 2. Evaluation of the cryo-EM map quality. a, FSC curves of the overall PSI-FCPI (red) and peripheral FCPI (blue) calculated between independently refined half maps of the reconstruction. **b,** Angular distribution of the particles used for reconstruction of overall PSI-FCPI and peripheral FCPI. Each cylinder represents one view and the height of the cylinder is proportional to the number of particles for that view. **c,** Local resolution maps of the overall PSI-FCPI. **d,** Local resolution maps of the peripheral FCPI.

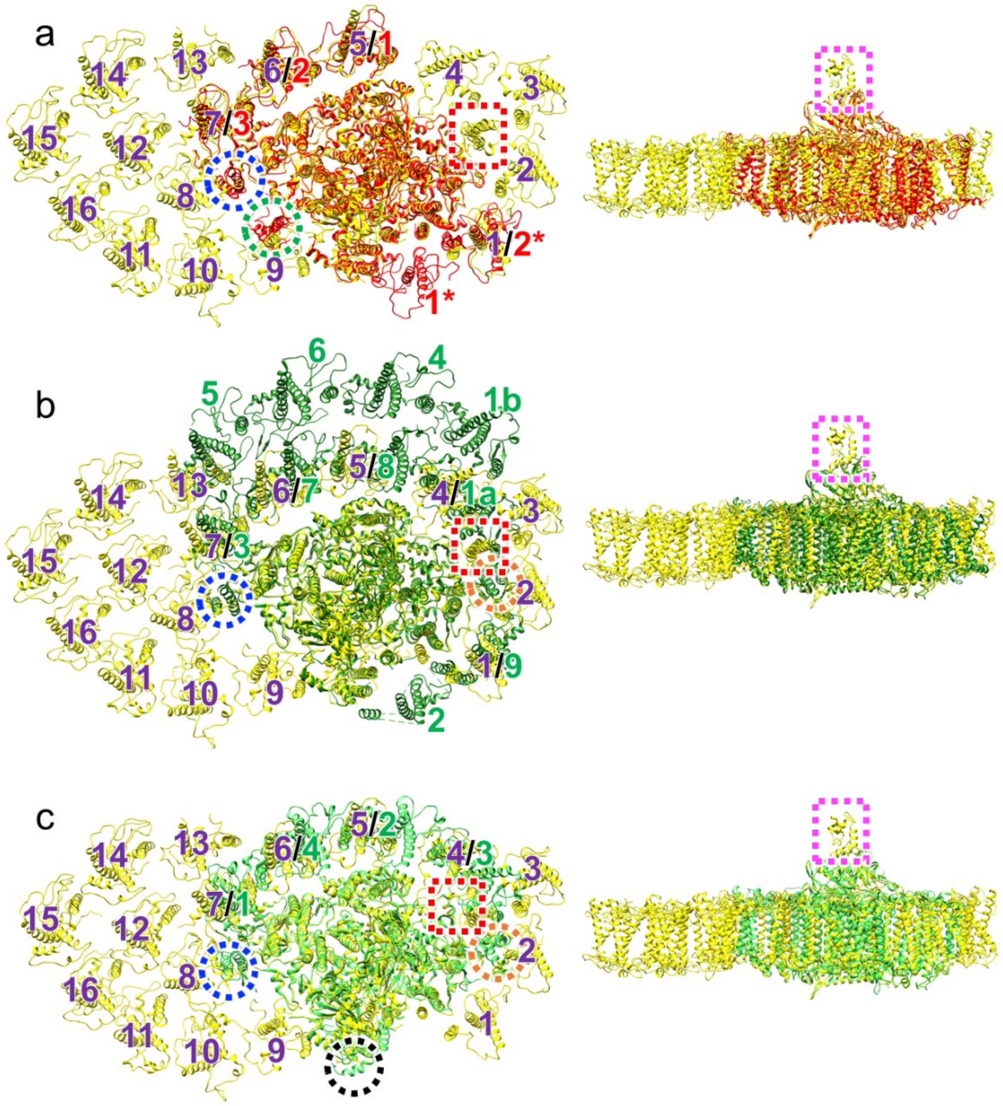

Supplementary Fig. 3. Superposition of the PSI-FCPI structure with other PSI-LHC structures. Overall superposed structures of the PSI-LHC supercomplexes with a view from the stromal side (left) and from a direction parallel to the membrane plane (right). PsaG, PsaH, PsaK and PsaO, which are absent in the diatom PSI-FCPI structure, are indicated by brown, black, blue and green dashed circles, respectively. Psa28 and Unknown1, which are found in the diatom PSI-FCPI structure only, are indicated by red and magenta dashed squares, respectively. The numbering 1–16 (purple) stands for Fcpa1–16 subunits, respectively. **a,** Diatom PSI-FCPI (yellow) vs. red algal PSI-LHCR (red; PDB: 5ZGB). Letters of 1–3, 1* and 2* (red) indicate Lhcr1–3, Lhcr1* and Lhcr2*, respectively. **b,** Diatom PSI-FCPI (yellow) vs. green algal PSI-LHCI (forest green; PDB: 6JO5). Letters of 1a, 1b and 2–9 (forest green) indicate Lhca1a, Lhca1b and Lhca2–9, respectively. **c,** Diatom PSI-FCPI (yellow) vs. plant PSI-LHCI (green; PDB: 5L8R). The numbering 1–4 (green) stands for Lhca1–4, respectively.

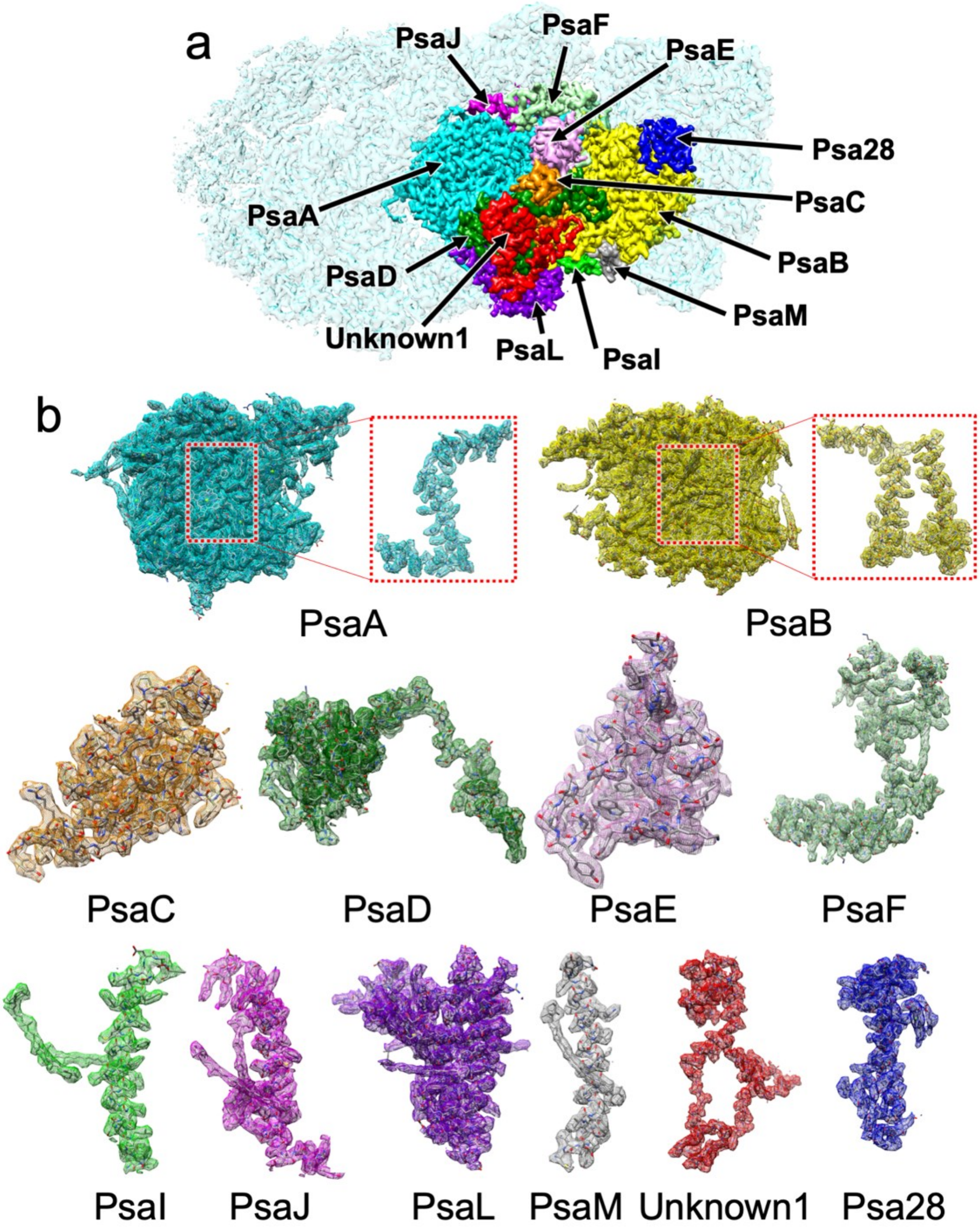

Supplementary Fig. 4. Cryo-EM density maps and structures of the PSI core subunits. a, The positions of each PSI core subunit with a view from the stromal side. **b,** Superposition of the densities with each PSI core subunit. The densities for each subunit of the PSI core are shown as meshes, and the corresponding models are shown as gray sticks. Red boxes indicate expanded views for a part of the individual PsaA and PsaB subunits. The amino-acid sequences of the PSI subunits are taken from the accession numbers of DDBJ/EMBL/Genbank: psaA,LC509439; psaB, LC509440; psaC, LC509441; psaD, LC509442; psaE, LC509443; psaF, LC509444; psaI, LC509445; psaJ, LC509446; psaL,LC509447;psaM, LC509448; psa28, LC509449.

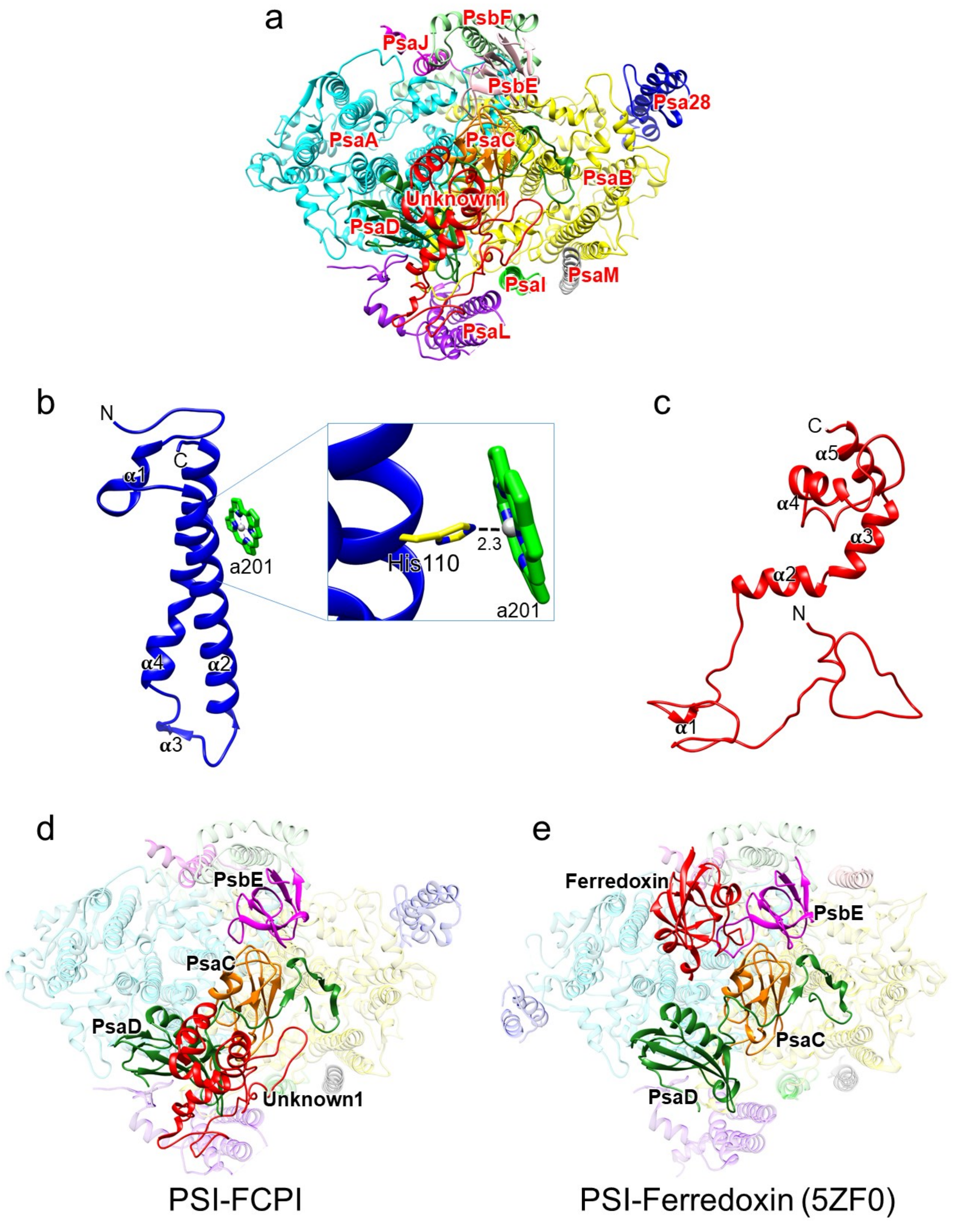

Supplementary Fig. 5. Locations and structures of the two novel subunits in the diatom PSI core, and comparison with that of ferredoxin. a, The positions of each PSI core subunit with a view from the stromal side. **b,** The structure of Psa28, with an expanded view of Chl *a* and its ligand of His110. **c,** The structure of Unknown1. **d,** The binding site of Unknown1 in the PSI-FCPI with a view from the stromal side. **e,** The binding site of ferredoxin in a cyanobacterial PSI-ferredoxin complex with a view from the stromal side (PDB: 5ZF0).

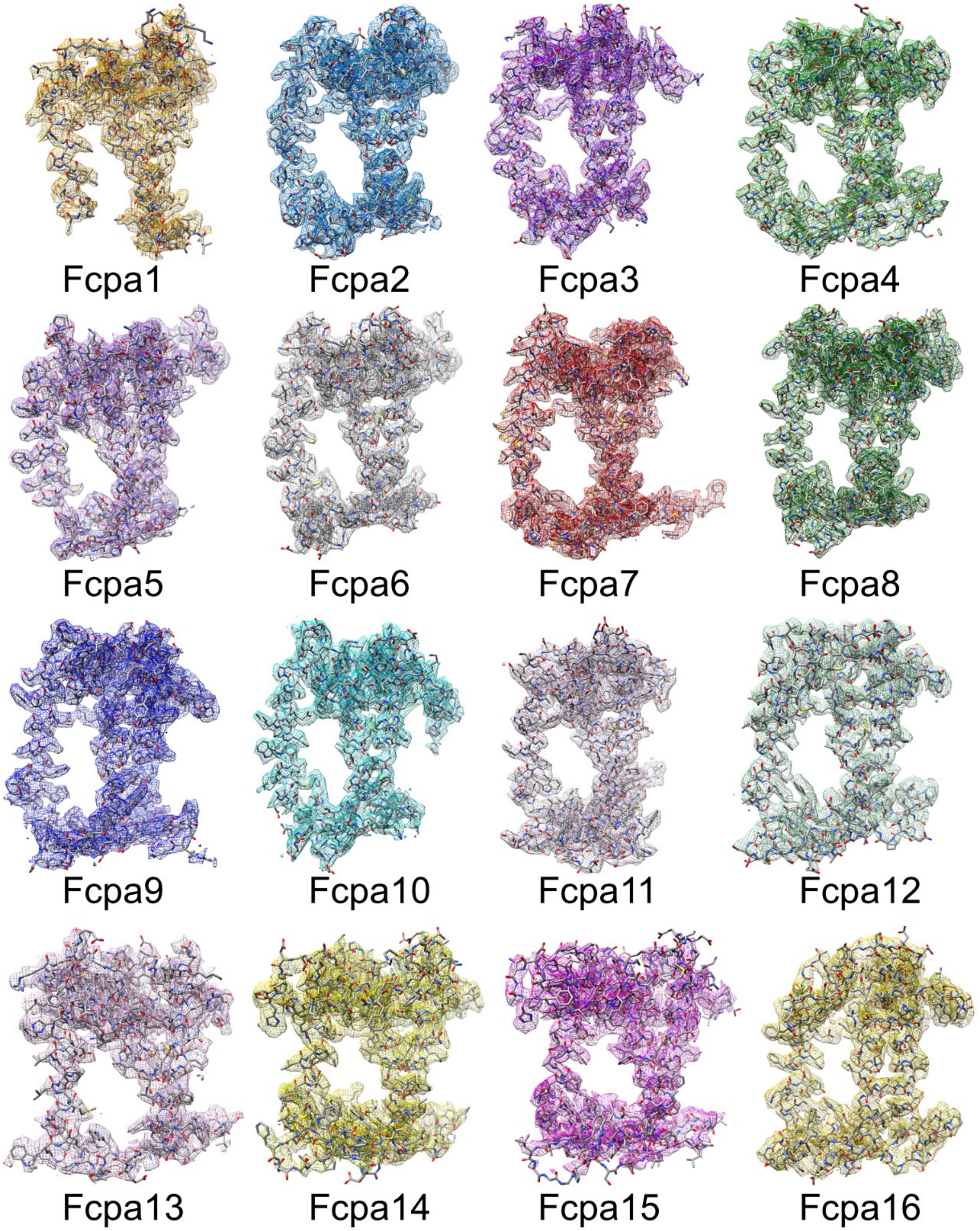

Fcpa1
Fcpa2
Fcpa3
Fcpa4
Fcpa5
Fcpa6
Fcpa7
Fcpa8
Fcpa9
Fcpa10
Fcpa11
Fcpa12
Fcpa13
Fcpa14
Fcpa15
Fcpa16

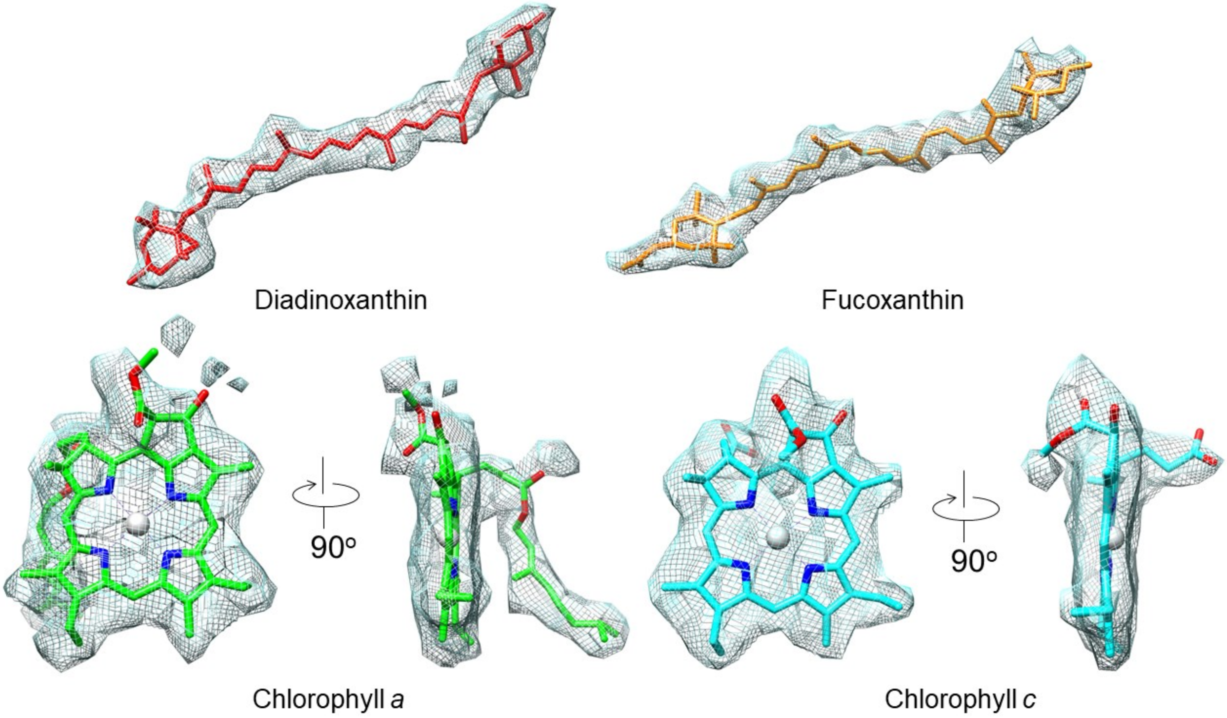

Supplementary Fig. 6. Cryo-EM density maps and structures of the 16 FCPI subunits and pigment molecules. The densities for each FCPI subunit and pigment molecules are shown as meshes and the corresponding models are shown as sticks.

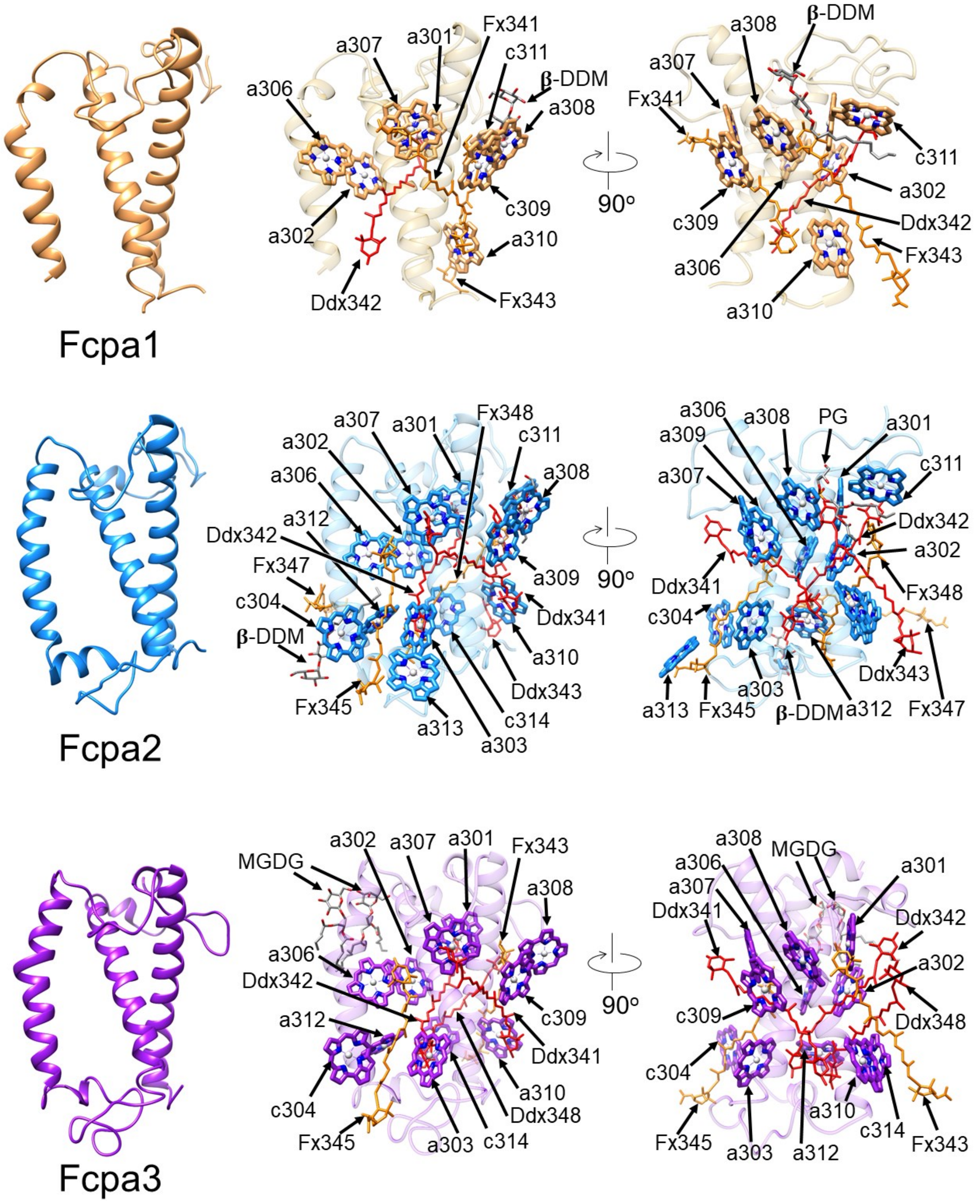

Fcpa1
a307
a301
Fx341
c311
β-DDM
a306
a308
a302
c309
a310
Ddx342
Fx343
90°
Fcpa2
Fx348
PG
Ddx342
Fx347
c304
β-DDM
Fx345
a313
c314
a303
a312
Ddx341
Ddx343
Fcpa3
MGDG
Ddx348

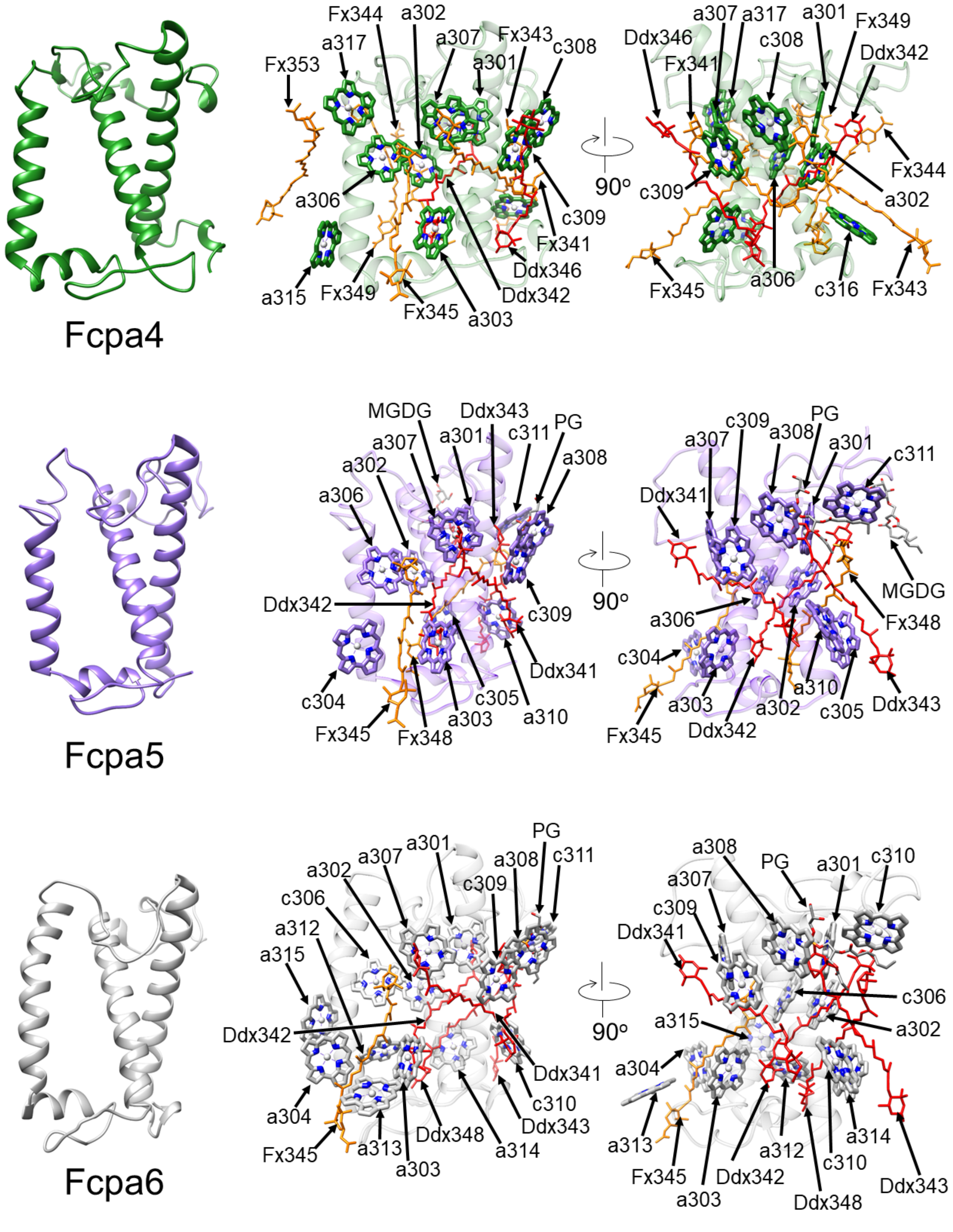
Fcpa4
Fx344
a302
a317
a307
Fx343
c308
Fx353
a301
a306
c309
Fx341
Ddx346
a315
Fx349
Fx345
Ddx342
a303
90°
a307
a317
a301
Fx349
Ddx346
c308
Ddx342
Fx341
Fx344
c309
a302
Fx345
a306
c316
Fx343
Fcpa5
MGDG
Ddx343
a307
a301
c311
PG
a302
a308
a306
Ddx342
c309
Ddx341
c304
c305
a310
Fx345
Fx348
a303
90°
c309
PG
a307
a308
a301
c311
Ddx341
MGDG
a306
Fx348
c304
a310
a303
a302
c305
Ddx343
Fx345
Ddx342
Fcpa6
PG
a301
a307
a302
a308
c311
c306
c309
a312
a315
Ddx342
Ddx341
c310
a304
Ddx348
Ddx343
Fx345
a313
a314
a303
90°
a308
PG
a301
c310
a307
c309
Ddx341
c306
a315
a302
a304
a313
a314
a312
Fx345
c310
Ddx342
Ddx343
a303
Ddx348

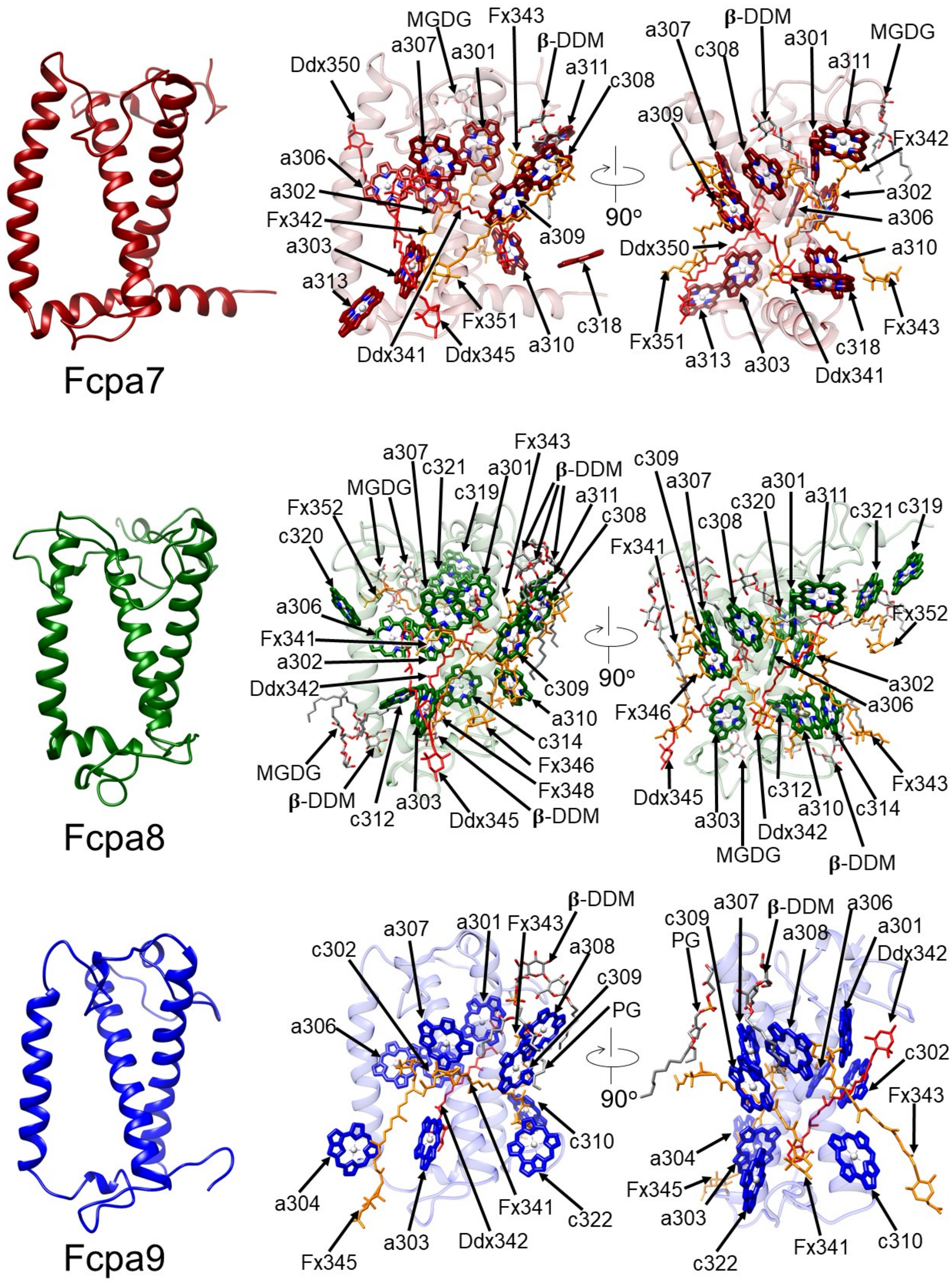

Fcpa7
Fcpa8
Fcpa9
90°

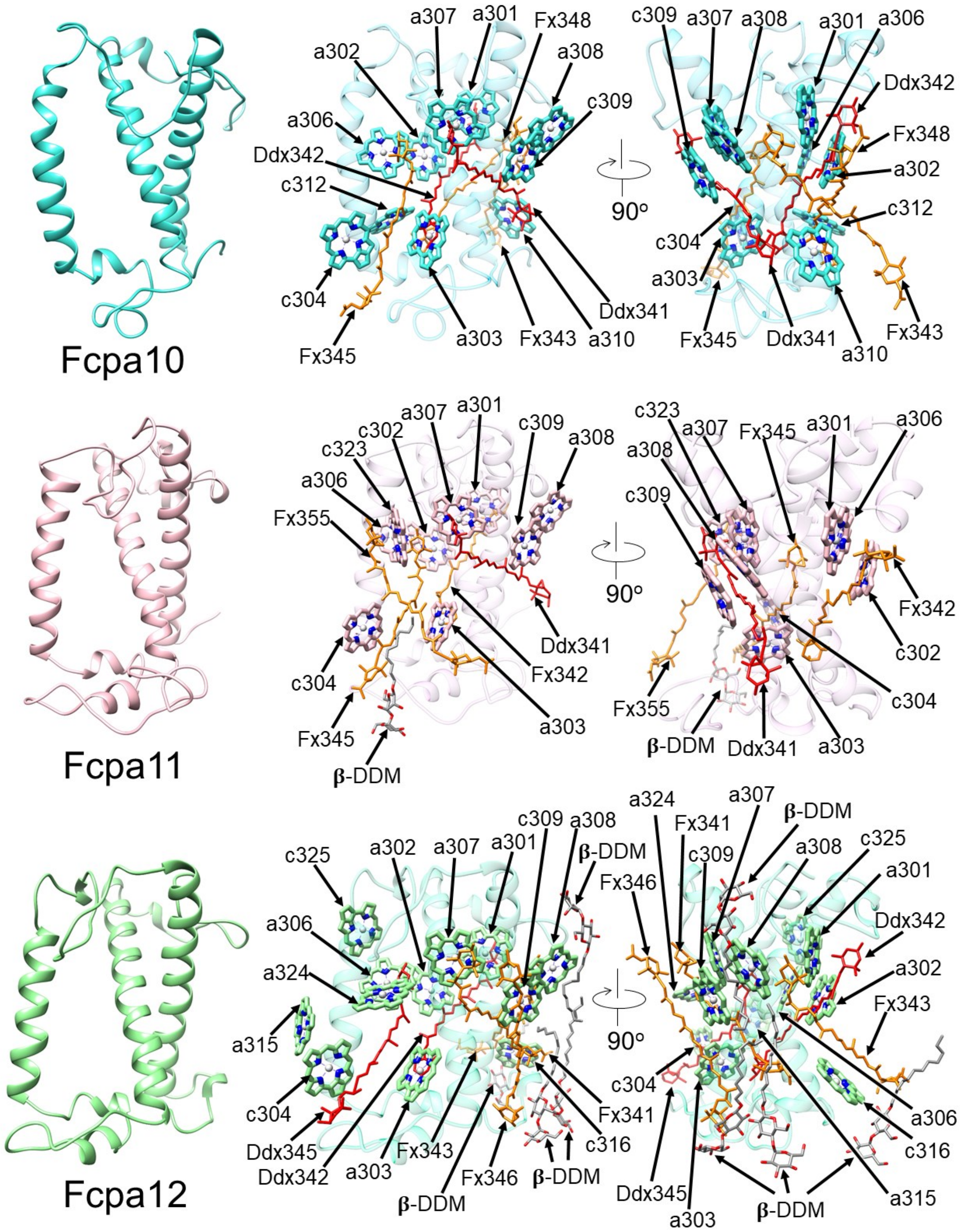
Fcpa10
a307
a301
Fx348
a302
a308
c309
a306
Ddx342
c312
c304
Fx345
a303
Fx343
a310
Ddx341
90°
c309
a307
a308
a301
a306
Ddx342
Fx348
a302
c312
c304
a303
Fx345
Ddx341
a310
Fx343
Fcpa11
a307
a301
c302
c309
a308
c323
a306
Fx355
c304
Fx345
β-DDM
Ddx341
Fx342
a303
90°
c323
a307
Fx345
a301
a306
a308
c309
Fx342
c302
c304
Fx355
β-DDM
Ddx341
a303
Fcpa12
c309
a308
c325
a302
a307
a301
β-DDM
a306
a324
a315
c304
Ddx345
Ddx342
a303
Fx343
β-DDM
Fx346
β-DDM
c316
Fx341
90°
a324
a307
β-DDM
Fx341
c309
a308
c325
Fx346
a301
Ddx342
a302
Fx343
c304
a306
c316
Ddx345
a303
β-DDM
a315

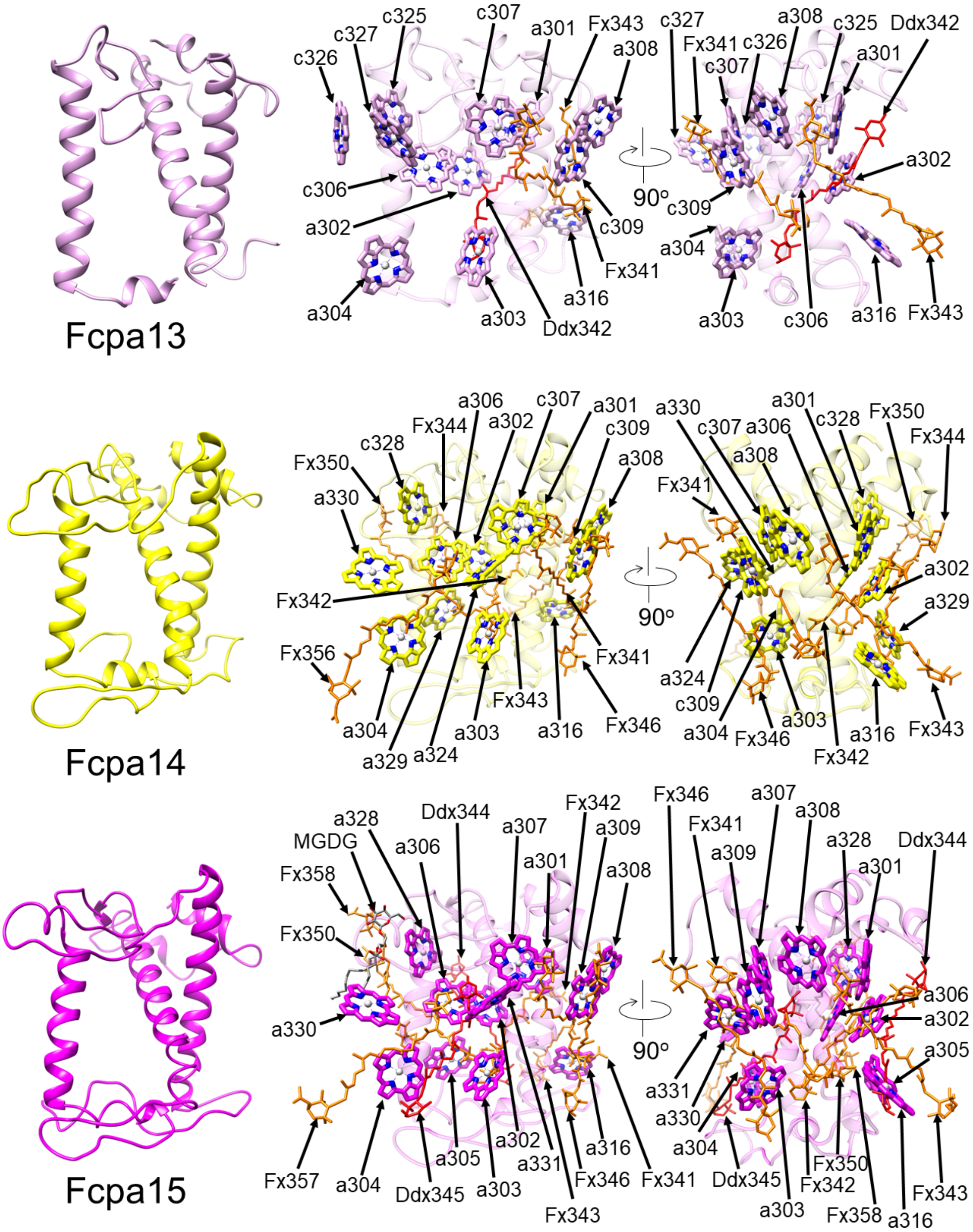
Fcpa13
c326
c327
c325
c307
a301
Fx343
a308
c306
a302
a304
a303
Ddx342
a316
Fx341
c309
90°
c327
Fx341
c326
c307
a308
c325
Ddx342
a301
a302
c309
a304
a303
c306
a316
Fx343
Fcpa14
a306
c307
a301
Fx344
a302
c309
c328
a308
Fx350
a330
Fx342
Fx356
a304
a329
a324
a303
Fx343
a316
Fx346
Fx341
90°
a330
c307
a306
a301
c328
Fx350
Fx344
a308
Fx341
a302
a329
a324
c309
a304
Fx346
a303
Fx342
a316
Fx343
Fcpa15
a328
Ddx344
MGDG
a306
a307
Fx342
a309
Fx358
a301
a308
Fx350
a330
Fx357
a304
Ddx345
a305
a303
a302
a331
Fx343
Fx346
a316
Fx341
90°
Fx346
a307
Fx341
a308
a309
a328
Ddx344
a301
a306
a302
a305
a331
a330
a304
Ddx345
a303
Fx350
Fx342
Fx358
a316
Fx343

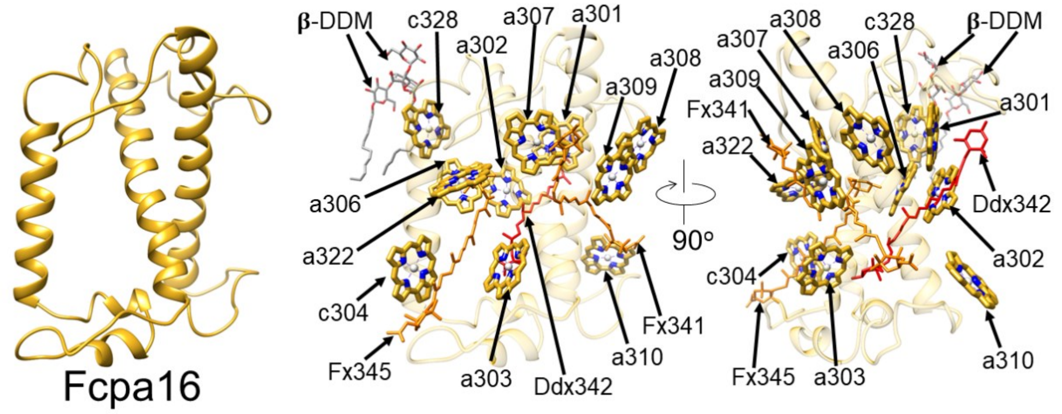

Supplementary Fig. 7. Structures of the individual Fcpa subunits. Structures of the apo-proteins of each Fcpa subunit (Fcpa1–16) are shown in the left-side, and arrangements of the pigments and co-factors in each Fcpa subunit viewed at the same directions as that in the left-side and at directions rotated 90° clockwise are shown in the middle and right-side, respectively.

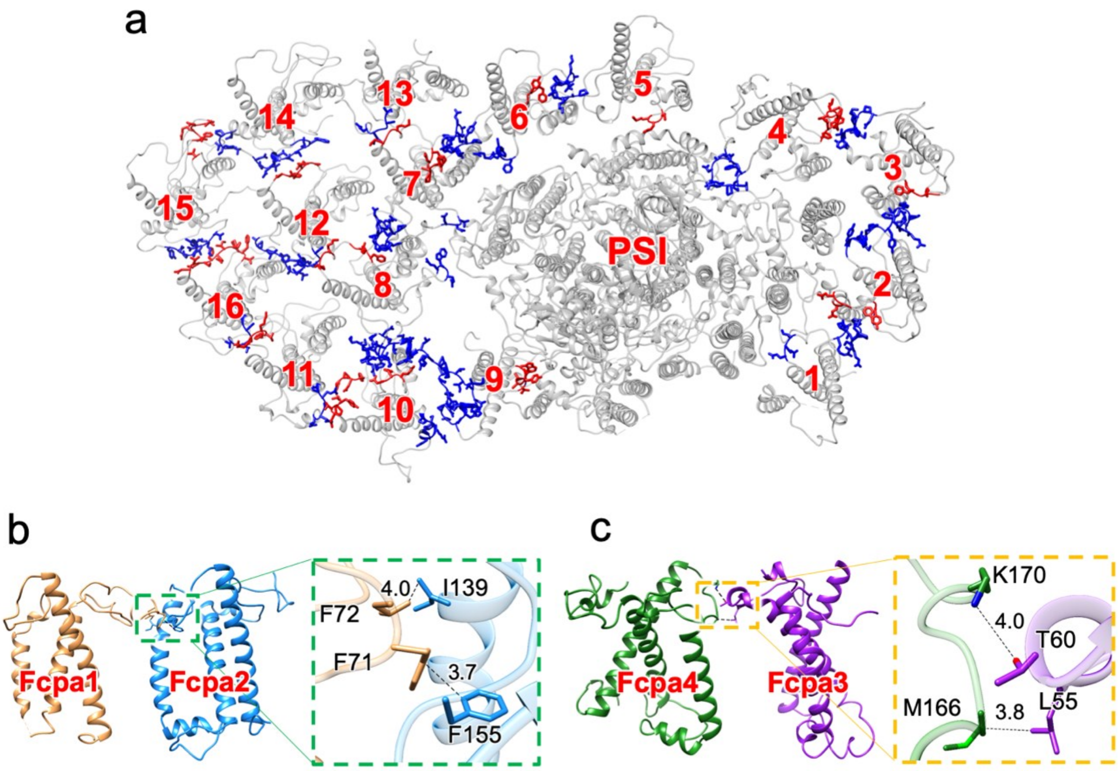

Supplementary Fig. 8. Protein-protein interactions among FCPI subunits. a, Interfaces among FCPI subunits with a view from the stromal side. The numbering 1–16 stands for Fcpa1–16 subunits, respectively. Blue and red sticks indicate characteristic loops. **b,** Interfaces between Fcpa1 and Fcpa2, with an expanded view (green dashed box). **c,** Interactions between Fcpa3 and Fcpa4, with an expanded view (orange dashed box). For clarity, only protein-protein interactions are shown, whereas the pigment-pigment and protein–pigment interactions are omitted. Interactions are indicated by dashed lines with distances labeled in Å.

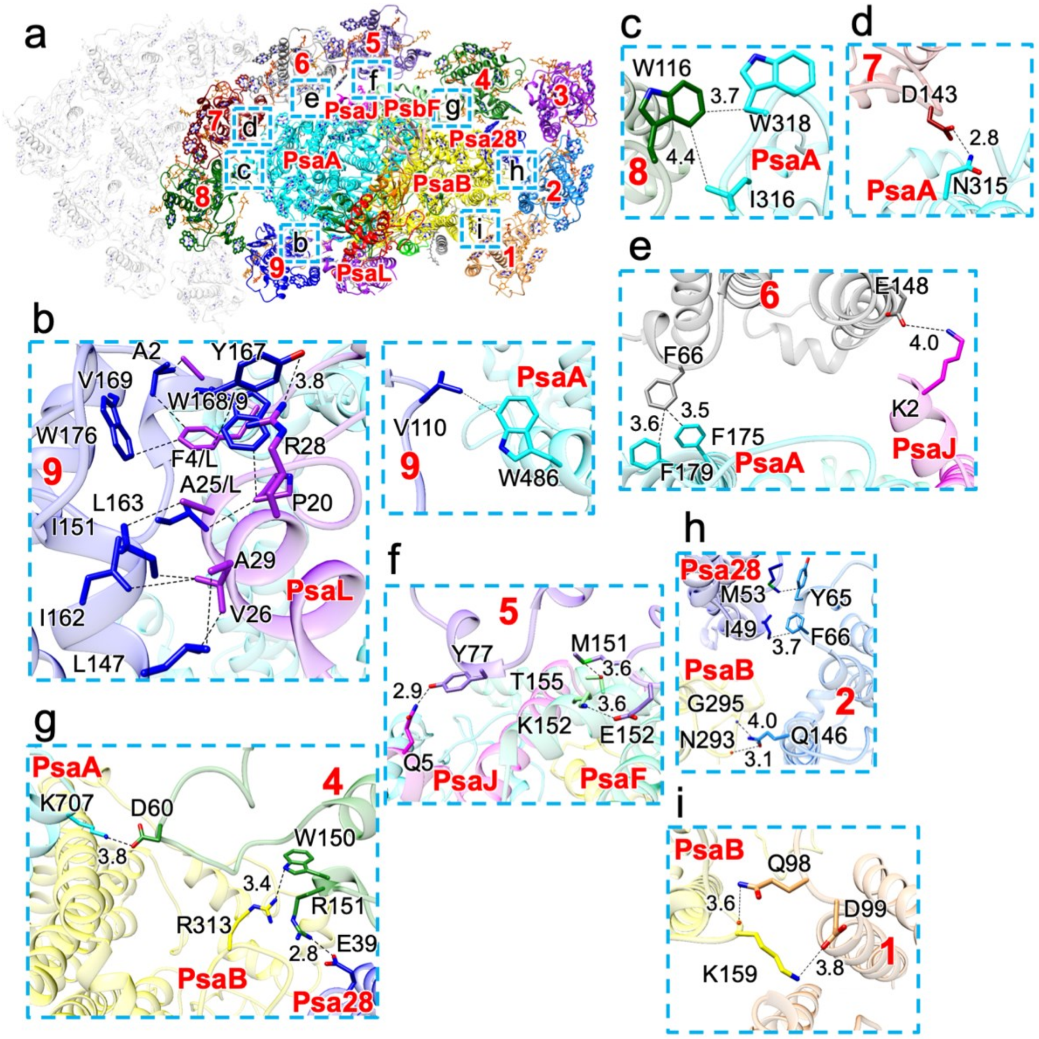

Supplementary Fig. 9. Protein-protein interactions between the inner FCPI ring subunits and the PSI core. a, Interactions between the inner FCPI ring subunits and PSI core with a view from the stromal side. Squared areas are enlarged in panels **(b–i)**. The numbering 1–9 stands for Fcpa1–9 subunits, respectively. **b,** Interactions between Fcpa9 and PsaL at the stromal side and between Fcpa9 and PsaA at the lumenal side. **c,** Interactions between Fcpa8 and PsaA. **d,** Interactions between Fcpa7 and PsaA. **e,** Interaction between Fcpa6 and PsaA/PsaJ. **f,** Interaction between Fcpa5 and PsaF/PsaJ. **g,** Interaction between Fcpa4 and PsaA/PsaB/Psa28. **h,** Interaction between Fcpa2 and PsaB/Psa28. **i,** Interaction between Fcpa1 and PsaB. For clarity, only protein-protein interactions are shown, whereas the pigment-pigment and protein–pigment interactions are omitted. Interactions are indicated by dashed lines with distances labeled in Å.

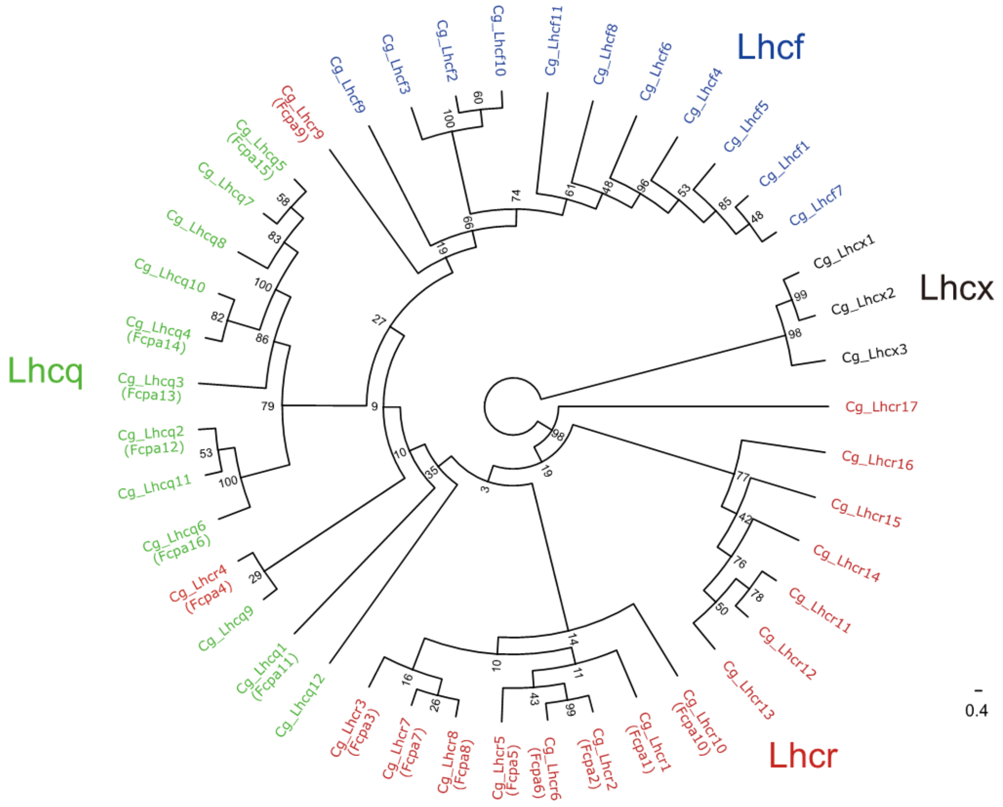

Supplementary Fig. 10. Phylogenetic tree of the *C. gracilis* LHC proteins (FCPs). A maximum likelihood tree rooted at the Lhcx clade is shown with the bootstrap values from 1,000 resamplings. Sequence information for the 16 FCPI subunits identified in the present PSI-FCPI structure is shown in Supplementary Table 5.

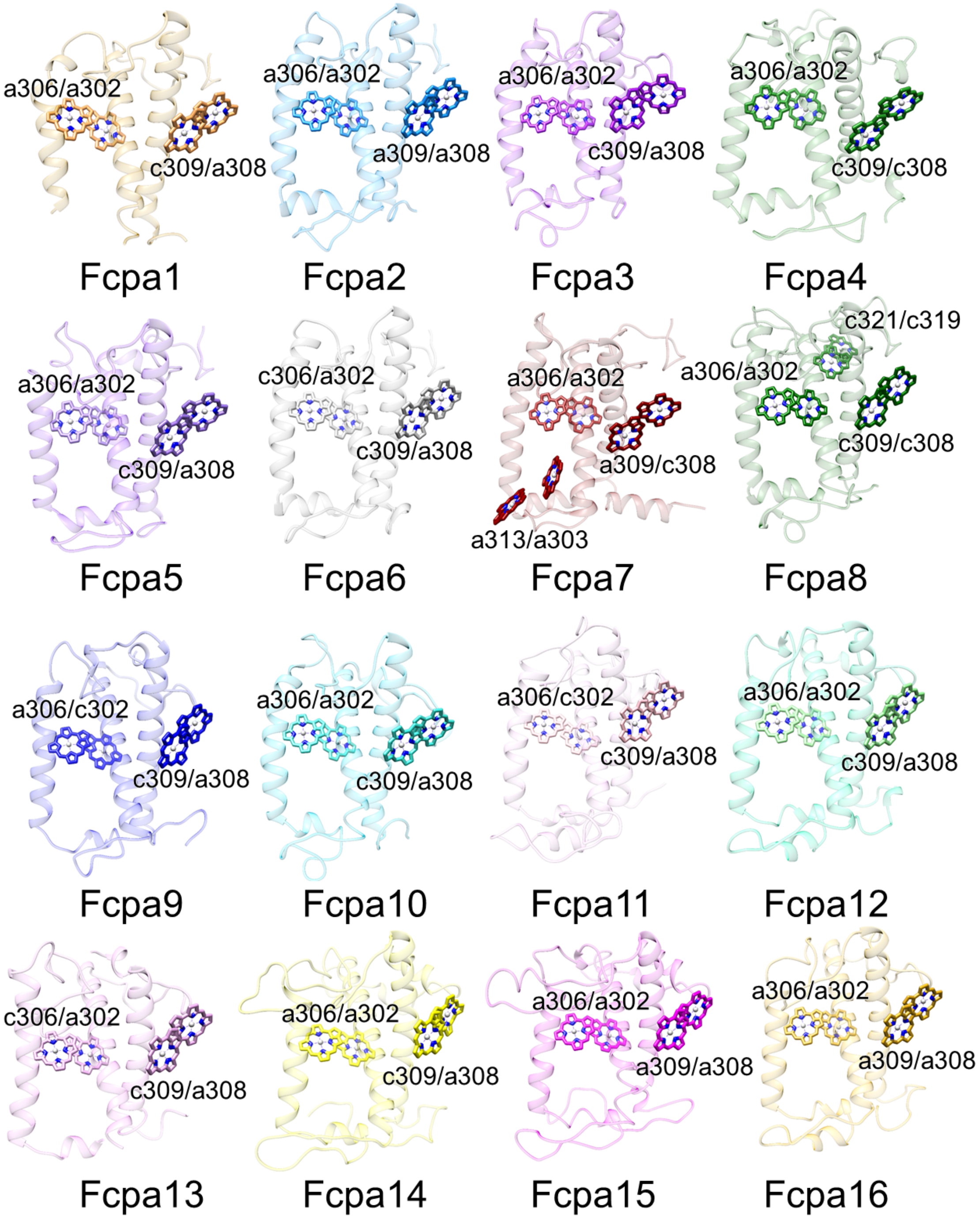

Supplementary Fig. 11. Dimeric Chls in each of the 16 Fcpa subunits. The structure is viewed from the side of the membrane.

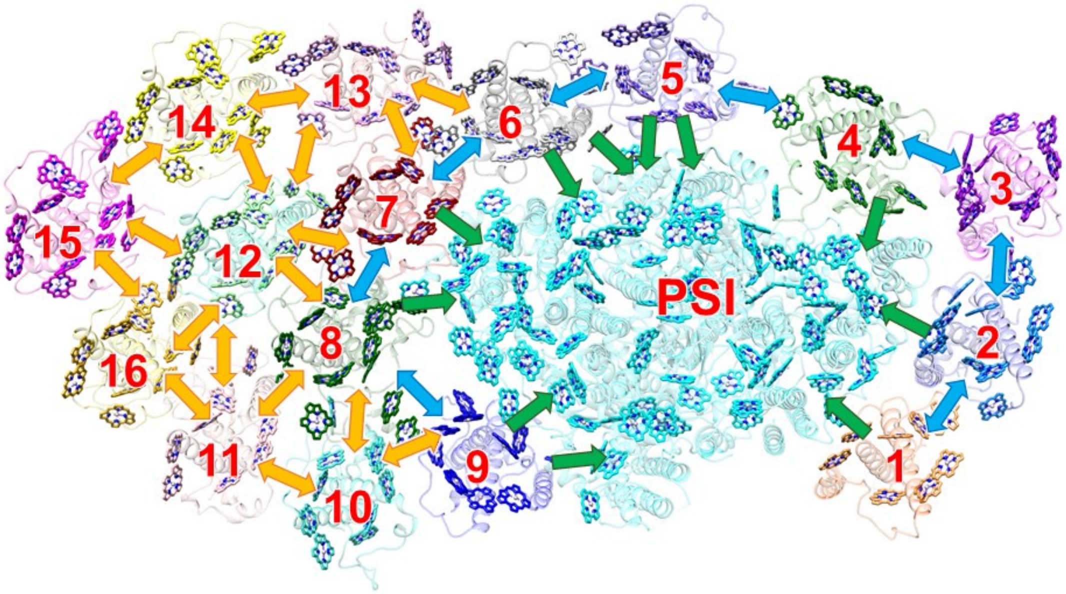

Supplementary Fig. 12. Possible excitation-energy transfer pathways in the PSI-FCPI. The structure is viewed from the stromal side. The numbering 1–16 stands for Fcpa1–16 subunits, respectively. Orange double-headed arrows indicate the pathways within the peripheral FCPIs and between the peripheral and ring FCPIs (see Fig. 3 for more detailed interactions). Blue double-headed arrows indicate the pathways within the inner FCPI ring (see Fig. 4 for more detailed interactions). Green arrows indicate the pathways between the inner FCPI ring and the PSI core (see Fig. 5 for more detailed interactions).

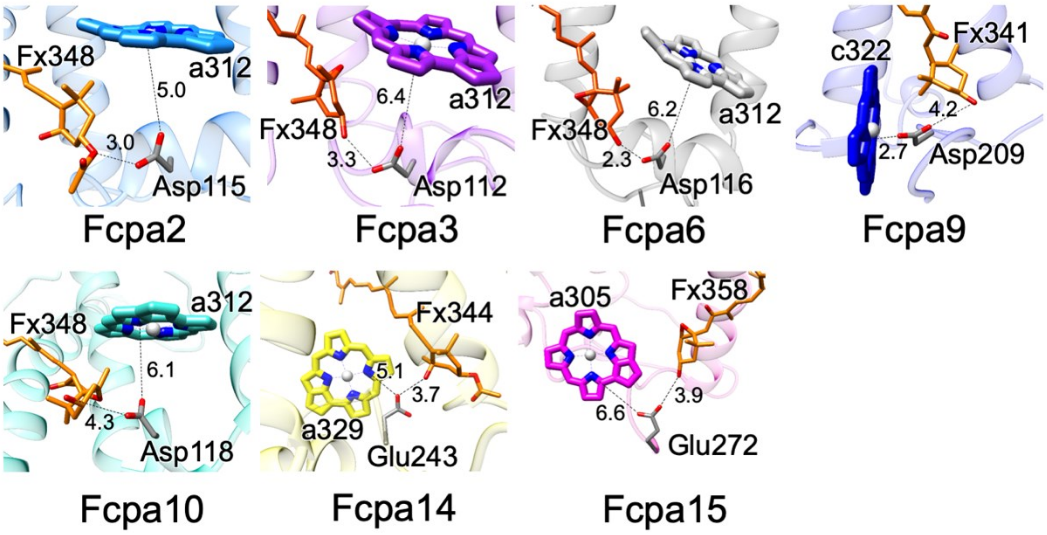

Supplementary Fig. 13. Possible pH-sensing sites for Chl-Car interactions near Asp/Glu residues at the lumenal side in the PSI-FCPI. Interactions between the Asp/Glu residues and pigments are indicated by black dashed lines with distances labeled in Å.

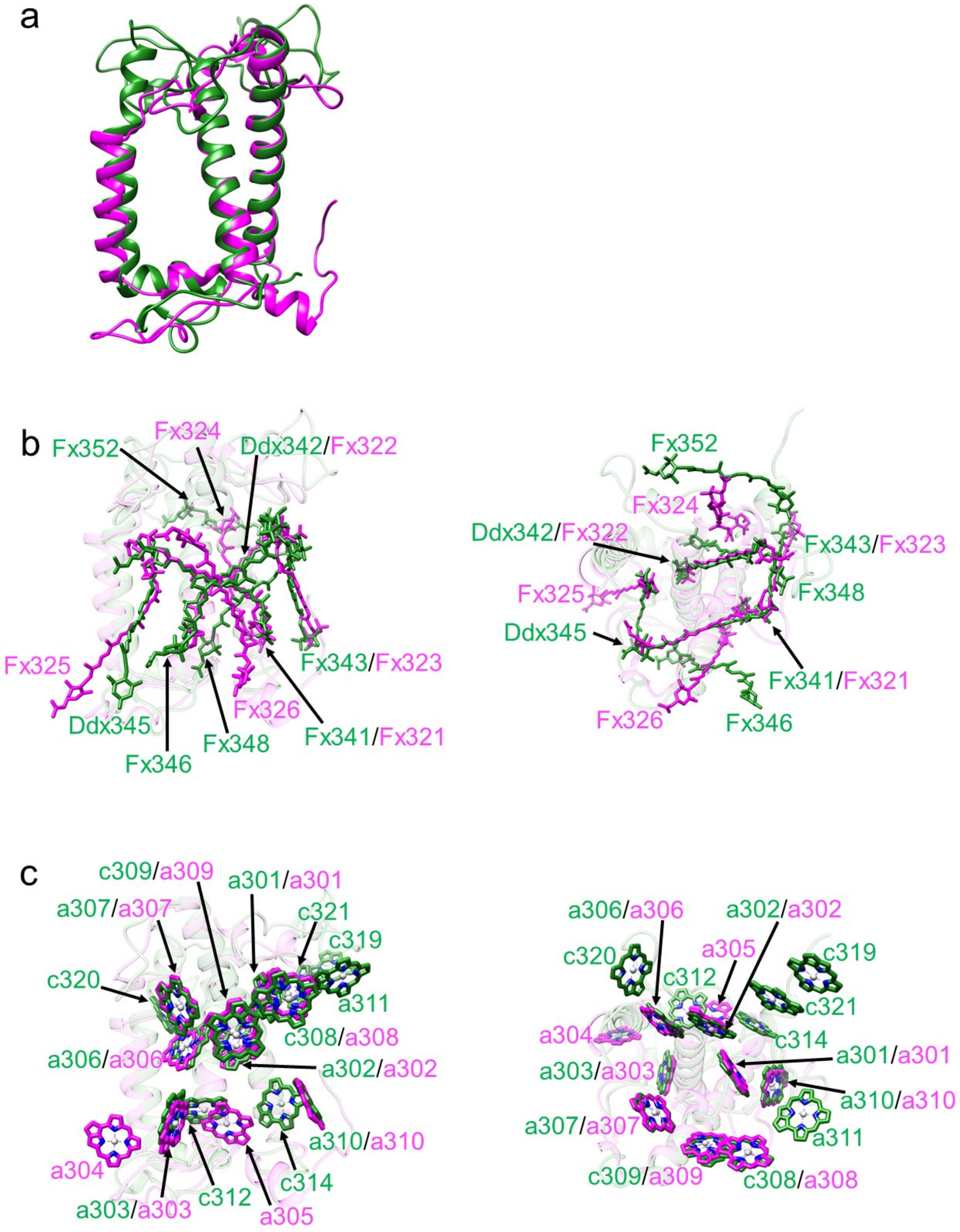

Supplementary Fig. 14. Structural comparisons of proteins (a), Chl-binding sites (b) and Car-binding sites (c) between Fcpa8 in the PSI-FCPI (green) and a protomer of the S-tetramer in the diatom PSII-FCPII (magenta) (PDB: 6J40). In panels **b** and **c**, the structures are superposed with views from a direction parallel to the membrane plane (left) and from the stromal side (right).

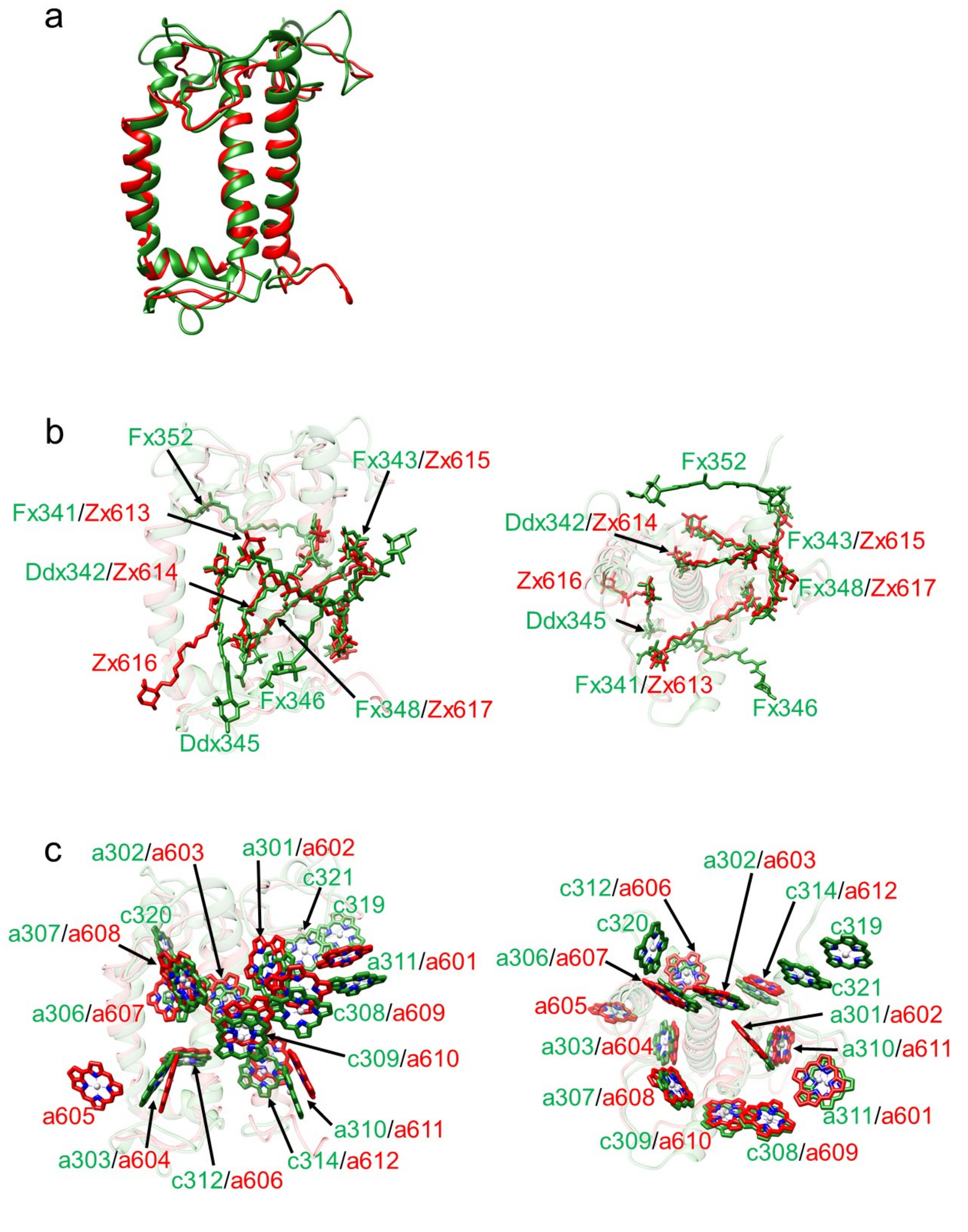

Supplementary Fig. 15. Structural comparisons of proteins (a), Chl-binding sites (b) and Car-binding sites (c) between Fcpa8 in the PSI-FCPI (green) and Lhcr1 in the red algal PSI-LHCI (red) (PDB: 5ZGB). In panels **b** and **c**, the structures are superposed with views from a direction parallel to the membrane plane (left) and from the stromal side (right). Zx indicates zeaxanthin.

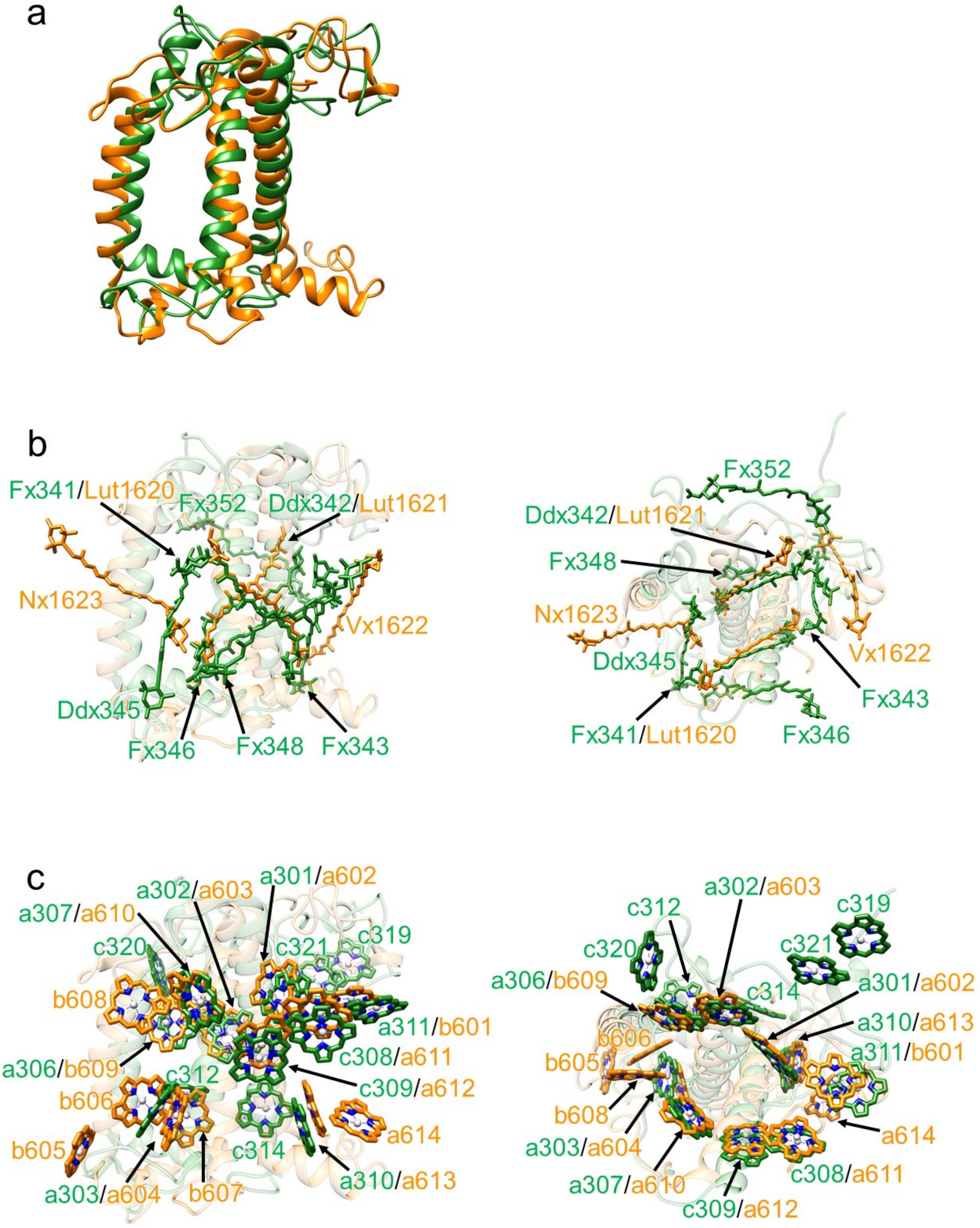

Supplementary Fig. 16. Structural comparisons of proteins (a), Chl-binding sites (b) and Car-binding sites (c) between Fcpa8 in the PSI-FCPI (green) and a protomer of the S-trimer in the plant PSII-LHCII (orange) (PDB: 5XNL). In panels **b** and **c**, the structures are superposed with views from a direction parallel to the membrane plane (left) and from the stromal side (right). Letters of Lut, Nx and Vx stand for lutein, neoxanthin and violaxanthin, respectively.

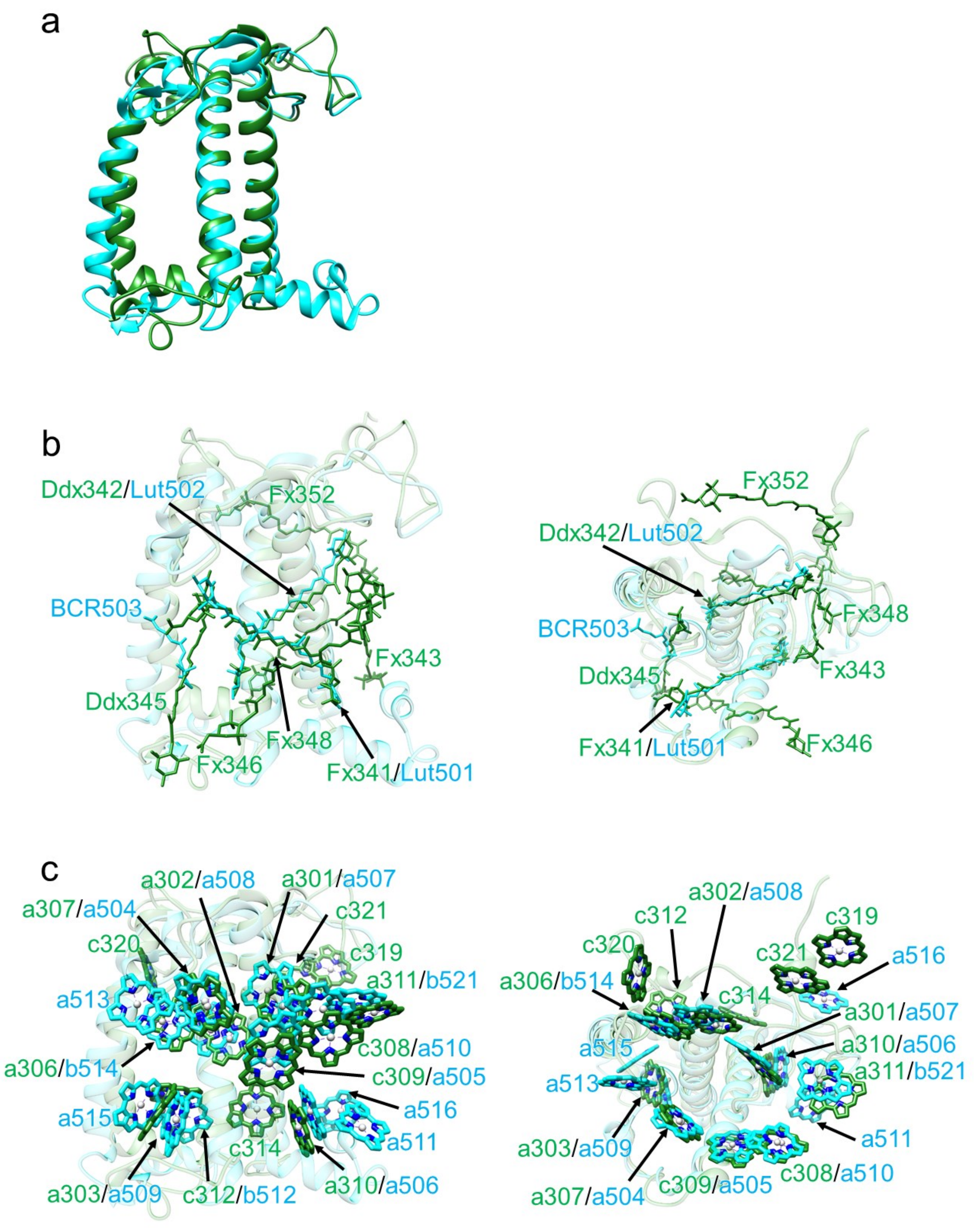

Supplementary Fig. 17. Structural comparisons of proteins (a), Chl-binding sites (b) and Car-binding sites (c) between Fcpa8 in the PSI-FCPI (green) and Lhca1 in the plant PSI-LHCI (cyan) (PDB: 5L8R). In panels **b** and **c**, the structures are superposed with views from a direction parallel to the membrane plane (left) and from the stromal side (right). Letters of Lut and BCR stand for lutein and β-carotene, respectively.

Supplementary Table 1. Cryo-EM data collection and structural analysis statistics.

Complex	peripheral FCPI	PSI-FCPI
PDB ID	6L4T	6L4U
EMDB ID	EMD-0834	EMD-0835
Data collection and processing		
Magnification	59000	59000
Voltage (kV)	300	300
Electron exposure (e^{-}/Å)	50	50
Defocus range (μm)	−1.5 to −3.0	−1.5 to −3.0
Pixel size (Å)	1.113	1.113
Symmetry imposed	C1	C1
Initial particle images (no.)	2689965	2689965
Final particle images (no.)	470801	470801
Map resolution (Å)	2.60	2.40
FSC threshold	0.143	0.143
Refinement		
Initial Model used (PDB code)	de novo	1JB0
Model resolution (Å)	2.6	2.4
FSC threshold	0.143	0.143
Map sharpening B factor ($Å^2$)	−74	−65
Model composition		
Non-hydrogen atoms	23863	62199
Protein	14209	40030
Ligand	9642	22013
Water	12	156
B factors ($Å^2$)		
Protein	68.3	46.1
Ligand	64.5	54.0
Water	44.7	35.3
R.m.s deviations		
Bond lengths (Å)	0.008	0.009
Bond angles (°)	2.039	1.923
Validation		
MolProbity score	1.78	1.92
Clashscore	5.31	5.76
Poor rotamers (%)	0.27	1.67
EMRinger score	3.52	4.06
Ramachandran plot		
Favored (%)	91.84	93.31
Allowed (%)	7.99	6.42
Disallowed (%)	0.16	0.27

Supplementary Table 2. Cofactors in each subunit of the PSI core.

Protein	Chorophyll	Carotenoid	Lipid		Others
			LHG	LMG	
**PsaA**	45 Chl *a*<br>1 Chl *a′*	5 BCR	2	1	1 [4Fe-4S] cluster,<br>1 phylloquinone
**PsaB**	41 Chl *a*	7 BCR	1	2	1 phylloquinone
**PsaC**					2 [4Fe-4S] cluster
**PsaD**					
**PsaE**					
**PsaF**	2 Chl *a*	1 BCR		1	
**PsaI**		2 BCR			
**PsaJ**	1 Chl *a*	2 BCR		1	
**PsaL**	3 Chl *a*	2 BCR			
**PsaM**		1 BCR			
**Psa28**	1 Chl *a*				
**Unknown1**					
**Total**	94	20	3	5	5

BCR, β-carotene; LMG, distearoylmonogalactosyl diglyceride; LHG, dipalmitoylphosphatidyl glycerol.

Supplementary Table 3. Cofactors in each subunit of the FCPI.

Protein	Chlorophyll		Carotenoid		Lipid	
	Chl *a*	Chl *c*	Fx	Ddx	LHG	LMG
**Fcpa1**	6	2	2	1		
**Fcpa2**	10	3	3	3	1	
**Fcpa3**	8	3	2	3		2
**Fcpa4**	7	3	6	2		
**Fcpa5**	7	4	2	3	1	1
**Fcpa6**	10	4	1	4	1	
**Fcpa7**	9	2	3	3		1
**Fcpa8**	7	7	5	2		3
**Fcpa9**	6	4	3	1	1	
**Fcpa10**	7	3	3	2		
**Fcpa11**	5	4	3	1		
**Fcpa12**	8	4	3	2		
**Fcpa13**	6	6	2	1		
**Fcpa14**	10	3	7			
**Fcpa15**	13		7	2		1
**Fcpa16**	9	2	2	1		
**Total**	128	54	54	31	4	8

Fx, fucoxanthin; Ddx, diadinoxanthin; LMG, distearoylmonogalactosyl diglyceride; LHG, dipalmitoylphosphatidyl glycerol.

Supplementary Table 4. Binding sites of pigments and their ligands in the FCPI subunits.

Chls[a]	Pigments (ligand) in each Fcpa				
	Fcpa1	Fcpa2	Fcpa3	Fcpa4	Fcpa5
301	Chl *a* (E107)	Chl *a* (E75)	Chl *a* (E72)	Chl *a* (E76)	Chl *a* (E87)
302	Chl *a* (H110)	Chl *a* (H78)	Chl *a* (H75)	Chl *a* (H79)	Chl *a* (H90)
303		Chl *a* (H_2O)	Chl *a* (-)	Chl *a* (-)	Chl *a* (Q104)
304		Chl *c* (Q125)	Chl *c* (Q122)		Chl *c* (Q132)
305					Chl *c* (H204)
306	Chl *a* (E162)	Chl *a* (E134)	Chl *a* (D131)	Chl *a* (E139)	Chl *a* (E141)
307	Chl *a* (E200)	Chl *a* (E178)	Chl *a* (E172)	Chl *a* (E184)	Chl *a* (E188)
308	Chl *a* (H_2O)	Chl *a* (LHG)	Chl *a* (F45)[b]	Chl *c* (E41)	Chl *a* (LHG)
309	Chl *c* (H203)	Chl *a* (N181)	Chl *c* (N175)	Chl *c* (N187)	Chl *c* (N191)
310	Chl *a* (Q217)	Chl *a* (Q195)	Chl *a* (Q189)		Chl *a* (H_2O)
311	Chl *c* (S68)[b]	Chl *c* (A38)[b]			Chl *c* (A50)[b]
312		Chl *a* (H114)	Chl *a* (H111)		
313		Chl *a* (Y99)[b]			
314		Chl *c* (H_2O)	Chl *c* (H_2O)		
315				Chl *a* (-)	
316				Chl *c* (S207)[b]	
317				Chl *a* (E141)	
**Cars**[a]					
341	Fx	Ddx	Ddx	Fx	Ddx
342	Ddx	Ddx	Ddx	Ddx	Ddx
343	Fx	Ddx	Fx	Fx	Ddx
344				Fx	
345		Fx	Fx	Fx	Fx
346				Ddx	
347		Fx			
348		Fx	Ddx		Fx
349				Fx	
353				Fx	

[a]Numbering of Chl and Car molecules in the PSI-FCPI.
[b]Mg coordination by a backbone carbonyl oxygen atom.

Chls[a]	Pigments (ligand) in each Fcpa				
	Fcpa6	Fcpa7	Fcpa8	Fcpa9	Fcpa10
301	Chl *a* (E76)	Chl *a* (E155)	Chl *a* (E131)	Chl *a* (E89)	Chl *a* (E78)
302	Chl *a* (H79)	Chl *a* (H158)	Chl *a* (H134)	Chl *c* (H92)	Chl *a* (H81)
303	Chl *a* (H_2O)	Chl *a* (H_2O)	Chl *a* (H_2O)	Chl *a* (-)	Chl *a* (H_2O)
304	Chl *a* (Q126)			Chl *a* (Q136)	Chl *c* (Q126)
305					
306	Chl *c* (E135)	Chl *a* (E215)	Chl *a* (E195)	Chl *a* (E145)	Chl *a* (E135)
307	Chl *a* (E180)	Chl *a* (E258)	Chl *a* (E240)	Chl *a* (E183)	Chl *a* (E178)
308	Chl *a* (LHG)	Chl *c* (H_2O)	Chl *c* (H_2O)	Chl *a* (H_2O)	Chl *a* (E45)
309	Chl *c* (N183)	Chl *a* (N261)	Chl *c* (N243)	Chl *c* (N186)	Chl *c* (N181)
310	Chl *c* (Q197)	Chl *a* (Q275)	Chl *a* (Q257)	Chl *c* (-)	Chl *a* (-)
311	Chl *c* (A39)[b]	Chl *a* (S113)[b]	Chl *a* (A75)[b]		
312	Chl *a* (H115)		Chl *c* (H175)		Chl *c* (H117)
313	Chl *a* (Y100)[b]	Chl *a* (P179)[b]			
314	Chl *a* (H_2O)		Chl *c* (H_2O)		
315	Chl *a* (-)				
316					
317					
318		Chl *c* (-)			
319			Chl *c* (Chl c321)		
320			Chl *c* (I111)[b]		
321			Chl *c* (H_2O)		
322				Chl *c* (D209)	
**Cars**[a]					
341	Ddx	Ddx	Fx	Fx	Ddx
342	Ddx	Fx	Ddx	Ddx	Ddx
343	Ddx	Fx	Fx	Fx	Fx
345	Fx	Ddx	Ddx	Fx	Fx
346			Fx		
348	Ddx		Fx		Fx
350		Ddx			
351		Fx			
352			Fx		

[a]Numbering of Chl and Car molecules in the PSI-FCPI.
[b]Mg coordination by a backbone carbonyl oxygen atom.

	Pigments (ligand) in each Fcpa					
**Chls**[a]	**Fcpa11**	**Fcpa12**	**Fcpa13**	**Fcpa14**	**Fcpa15**	**Fcpa16**
301	Chl *a* (E72)	Chl *a* (E66)	Chl *a* (E99)	Chl *a* (E84)	Chl *a* (E105)	Chl *a* (E80)
302	Chl *c* (H75)	Chl *a* (H69)	Chl *a* (H102)	Chl *a* (H87)	Chl *a* (H108)	Chl *a* (H83)
303	Chl *a* (H_2O)	Chl *a* (Q83)	Chl *a* (-)	Chl *a* (Q101)	Chl *a* (Q122)	Chl *a* (Q97)
304	Chl *c* (Q147)	Chl *c* (Q121)	Chl *a* (Q153)	Chl *a* (Q139)	Chl *a* (Q159)	Chl *c* (Q135)
305					Chl *a* (-)	
306	Chl *a* (E156)	Chl *a* (E130)	Chl *c* (E162)	Chl *a* (E148)	Chl *a* (E168)	Chl *a* (E144)
307	Chl *a* (E200)	Chl *a* (E171)	Chl *c* (E214)	Chl *c* (E206)	Chl *a* (E229)	Chl *a* (E185)
308	Chl *a* (E44)	Chl *a* (H_2O)	Chl *a* (E73)	Chl *a* (-)	Chl *a* (-)	Chl *a* (-)
309	Chl *c* (N203)	Chl *c* (N174)	Chl *c* (N217)	Chl *c* (N209)	Chl *a* (N232)	Chl *a* (N188)
310						Chl *a* (S208)[b]
312						
315		Chl *a* (H_2O)				
316		Chl *c* (S194)[b]	Chl *a* (-)	Chl *a* (S229)[b]	Chl *a* (-)	
322						Chl *a* (F165)[b]
323	Chl *c* (-)					
324		Chl *a* (F151)[b]		Chl *a* (-)		
325		Chl *c* (E134)	Chl *c* (E166)			
326			Chl *c* (H164)			
327			Chl *c* (P190)[b]			
328				Chl *c* (E152)	Chl *a* (E172)	Chl *c* (E148)
329				Chl *a* (M245)[b]		
330				Chl *a* (H178)	Chl *a* (H202)	
331					Chl *a* (-)	
**Cars**[a]						
341	Ddx	Fx	Fx	Fx	Fx	Fx
342	Fx	Ddx	Ddx	Fx	Fx	Ddx
343		Fx	Fx	Fx	Fx	
344				Fx	Ddx	
345	Fx	Ddx			Ddx	Fx
346		Fx		Fx	Fx	
350				Fx	Fx	
355	Fx					
356				Fx		
357					Fx	
358					Fx	

[a]Numbering of Chl and Car molecules in the PSI-FCPI.

[b]Mg coordination by a backbone carbonyl oxygen atom.

Supplementary Table 5. Fcpa subunits identified in the present PSI-FCPI structure and comparisons of each Fcpa subunits with Fcpa8.

Protein	Gene	Gene ID[a]	Identity (%)[b]	Similarity (%)[b]	RMSD (Å) / Aligned Cα atoms[c]
**Fcpa1**	*Lhcr1*	LC508706	32.9	44.4	1.71 / 137
**Fcpa2**	*Lhcr2*	LC508707	37.6	54.0	1.24 / 160
**Fcpa3**	*Lhcr3*	LC508708	36.3	51.0	1.36 / 146
**Fcpa4**	*Lhcr4*	LC508709	26.9	38.5	2.43 / 140
**Fcpa5**	*Lhcr5*	LC508710	27.5	39.3	1.43 / 160
**Fcpa6**	*Lhcr6*	LC508711	42.3	52.6	1.10 / 161
**Fcpa7**	*Lhcr7*	LC508712	33.6	48.3	1.75 / 164
**Fcpa8**	*Lhcr8*	LC508713	100	100	0.00 / 213
**Fcpa9**	*Lhcr9*	LC508714	32.8	44.8	1.83 / 139
**Fcpa10**	*Lhcr10*	LC508715	35.5	49.4	1.44 / 151
**Fcpa11**	*Lhcq1*	LC508716	24.2	34.1	2.47 / 144
**Fcpa12**	*Lhcq2*	LC508717	30.8	39.5	2.15 / 136
**Fcpa13**	*Lhcq3*	LC508718	31.9	44.0	1.65 / 108
**Fcpa14**	*Lhcq4*	LC508719	23.8	39.7	2.13 / 133
**Fcpa15**	*Lhcq5*	LC508720	24.7	36.3	2.03 / 135
**Fcpa16**	*Lhcq6*	LC508721	27.0	39.7	2.08 / 133

[a]DDBJ/EMBL/Genbank accession number
[b]Identity and similarity are shown for the amino acid sequences between Fcpa8 and other 15 Fcpa subunits, and were estimated by an optimal global alignment of two sequences using EMBOSS needle (https://www.ebi.ac.uk/Tools/psa/emboss_needle/).
[c]RMSDs were calculated between the structure of Cα atoms of Fcpa8 and other Fcpa subunits.

Supplementary Table 6. Correspondence of numbering of pigments described in the text with those in the PDB file for each FCPI subunit.

Chls in the text	PDB No. (Chain ID)				
	Fcpa1	Fcpa2	Fcpa3	Fcpa4	Fcpa5
301	301 (1)	303 (2)	301 (3)	302 (4)	302 (5)
302	302 (1)	304 (2)	302 (3)	303 (4)	303 (5)
303		305 (2)	303 (3)	304 (4)	304 (5)
304		306 (2)	304 (3)		305 (5)
305					306 (5)
306	303 (1)	307 (2)	305 (3)	305 (4)	307 (5)
307	304 (1)	308 (2)	306 (3)	306 (4)	308 (5)
308	305 (1)	309 (2)	307 (3)	307 (4)	309 (5)
309	306 (1)	310 (2)	308 (3)	308 (4)	310 (5)
310	307 (1)	311 (2)	309 (3)		311 (5)
311	308 (1)	312 (2)			312 (5)
312		851 (B)	310 (3)		
313		313 (2)			
314		314 (2)	311 (3)		
315				309 (4)	
316				310 (4)	
317				311 (4)	
**Cars in the text**					
341	309 (1)	315 (2)	312 (3)	312 (4)	313 (5)
342	310 (1)	316 (2)	313 (3)	313 (4)	314 (5)
343	302 (2)	317 (2)	314 (3)	301 (5)	303 (6)
344				314 (4)	
345		318 (2)	315 (3)	315 (4)	315 (5)
346				316 (4)	
347		203 (2u)			
348		319 (2)	316 (3)		316 (5)
349				317 (4)	
353				205 (2u)	

Chls in the text	PDB No. (Chain ID)				
	Fcpa6	Fcpa7	Fcpa8	Fcpa9	Fcpa10
301	304 (6)	303 (7)	301 (8)	303 (9)	303 (10)
302	305 (6)	304 (7)	302 (8)	304 (9)	304 (10)
303	306 (6)	305 (7)	303 (8)	305 (9)	305 (10)
304	307 (6)			306 (9)	306 (10)
305					
306	308 (6)	306 (7)	304 (8)	307 (9)	307 (10)
307	309 (6)	307 (7)	305 (8)	308 (9)	308 (10)
308	310 (6)	308 (7)	306 (8)	309 (9)	309 (10)
309	311 (6)	309 (7)	307 (8)	310 (9)	310 (10)
310	312 (6)	310 (7)	308 (8)	311 (9)	311 (10)
311	313 (6)	311 (7)	309 (8)		
312	314 (6)		310 (8)		312 (10)
313	315 (6)	312 (7)			
314	316 (6)		311 (8)		
315	317 (6)				
318		313 (7)			
319			312 (8)		
320			313 (8)		
321			314 (8)		
322				312 (9)	
**Cars in the text**					
341	318 (6)	314 (7)	315 (8)	313 (9)	313 (10)
342	319 (6)	315 (7)	316 (8)	314 (9)	314 (10)
343	302 (7)	316 (7)	301 (10)	315 (9)	315 (10)
345	320 (6)	317 (7)	317 (8)	316 (9)	316 (10)
346			301 (11)		
348	321 (6)		318 (8)		317 (10)
350		318 (7)			
351		319 (7)			
352			302 (10)		

Chls in the text	PDB No. (Chain ID)					
	Fcpa11	Fcpa12	Fcpa13	Fcpa14	Fcpa15	Fcpa16
301	304 (11)	302 (12)	301 (13)	302 (14)	302 (15)	301 (16)
302	305 (11)	303 (12)	302 (13)	321 (12)	303 (15)	302 (16)
303	306 (11)	304 (12)	303 (13)	303 (14)	304 (15)	303 (16)
304	307 (11)	305 (12)	304 (13)	304 (14)	305 (15)	304 (16)
305					306 (15)	
306	308 (11)	306 (12)	305 (13)	305 (14)	307 (15)	305 (16)
307	309 (11)	307 (12)	306 (13)	306 (14)	308 (15)	306 (16)
308	310 (11)	308 (12)	307 (13)	307 (14)	309 (15)	307 (16)
309	311 (11)	309 (12)	308 (13)	308 (14)	310 (15)	308 (16)
310						309 (16)
315		310 (12)				
316		311 (12)	309 (13)	309 (14)	311 (15)	
322						310 (16)
323	312 (11)					
324		312 (12)		310 (14)		
325		313 (12)	310 (13)			
326			311 (13)			
327			312 (13)			
328				311 (14)	312 (15)	311 (16)
329				312 (14)		
330				313 (14)	313 (15)	
331					314 (15)	
**Cars in the text**						
341	313 (11)	314 (12)	313 (13)	314 (14)	315 (15)	312 (16)
342	314 (11)	315 (12)	314 (13)	315 (14)	316 (15)	313 (16)
343		316 (12)	315 (13)	316 (14)	317 (15)	
344				317 (14)	318 (15)	
345	315 (11)	317 (12)			319 (15)	314 (16)
346		301 (14)		318 (14)	320 (15)	
350				319 (14)	321 (15)	
355	316 (11)					
356				320 (14)		
357					322 (15)	
358					323 (15)	
